# Overemphasized “g”

**DOI:** 10.3390/jintelligence5040033

**Published:** 2017-10-01

**Authors:** Lazar Stankov

**Affiliations:** 1 The University of Sydney, Sydney 2006, Australia; lazondi@rocketmail.com; Tel.: +61-4-2697-0331; 2 The University of Southern Queensland, Toowoomba 4352, Australia

**Keywords:** general factor, “g”, complex problem solving, cognitive biases, emotional intelligence

## Abstract

In this paper I argue that the emphasis on “g” has become a hindrance to the study of broadly defined human cognitive abilities. Abilities captured by the first- and second-stratum factors in the Cattel-Horn-Carroll (CHC) theory have been neglected. The focus has been on a narrow range of cognitive processes that excludes those common to some sensory modalities and a host of new tasks and constructs that have become available through recent conceptual analyses and technological developments. These new areas have emerged from psychology itself (complex problem solving tasks and emotional intelligence) and from disciplines related to psychology like education and economics (economic games and cognitive biases in decision-making).

## 1. “g” on Top Versus Truncated Hierarchy

It is not widely known among researchers in human intelligence that there was a disagreement, mostly played out through private communications, between two proponents of the now popular CHC (Cattell-Horn-Carroll) theory [1,2,3]. Thus, John Horn and Jack Carroll had different views about the role of the “g” factor [4,5]. Horn did not accept the reasoning that led Carroll to place “g” at the top of the hierarchy and lend support for the views of Jensen [6]. Needless to say, Cattell was in a broad agreement with Horn (see [7]). Their preference was for the hierarchical structure with correlated broad second-order factors. These factors were always seen as being more informative and important than the general factor itself.

Over the years Horn has articulated both methodological—akin to Thurstone’s [8] points about primary mental abilities—and substantive arguments in defense of a truncated hierarchy. An example of the latter argued that since investment theory postulates the emergence of Gc from Gf due to the influences from non-cognitive processes (e.g., interests and self-beliefs; see [9]) whatever is captured by the Gc factor has to be different from Gf processes and, indeed, from the overarching “g” factor itself.

## 2. “g” Is Made to Look More Important than It Deserves

### 2.1. “g” Is Small

My own position is in-between. I believe that “g” does exist at the top of the hierarchy but its role is much smaller than what its ardent supporters wish to claim—it is a “diminutive general”, close to being negligible [10]. The emphasis on “g” has led to a relative neglect of the first- and second-stratum factors in the CHC theory. There are three reasons for adopting such a view. First, even if the investment theory as briefly outlined above operates in reality, one has to accept that traces of Gf remain within the Gc itself and therefore a “g” that is conceptually close to both Gf and Gc makes sense.

Second, starting with Spearman [11], it was assumed that positive manifold that is seen as being responsible for the emergence of “g” is characteristic of all cognitive processing. To put it the other way around: correlations between all cognitive tasks are positive. In my conversation with Cattell many years ago he defined “cognitive” as a reaction to stimuli that vary in terms of difficulty/complexity. Ever since that time I have used it as a rough-and-ready definition of the cognitive domain in studies of individual difference. To him, stimuli that vary in terms of their incentive values evoke non-cognitive process of motivation. As it happens, much of the work on intelligence was based on a biased selection of measures that tap “higher mental processes” (HMP) and very quickly sensory discrimination tasks (i.e., “peripheral processes” or “provincial powers”) were removed from consideration. Thus, HMP measures do not cover the whole domain of cognition and they are far from being a representative sample from that domain either [12] (p. 180). Clearly, a systematic exploration of individual differences in tasks from the population of all cognitive processes is needed. This is dictated not so much by a suspicion that some cognitive measures may correlate negatively (or have zero correlation) but rather by a need to fully explore the role of all cognitive processes in intelligence.

Third, our efforts to expand the coverage of cognition by studying individual differences in cognitive processes linked to sensory modalities other than vision (Gv), led us to a realization that some processes of importance for intelligent behavior are left out of consideration. For example, solving problems that require integration of elements spread over time—i.e., temporal integration tasks [13]—is better captured by the auditory (Ga) than by the visual tests [14]. Olfactory modality is important because of its links to memory [15] and tactile/kinaesthetic processes, while distinct from measures of intelligence, are undoubtedly important in problem-solving tasks called for in sport and manual work [16]. Thus, broad olfactory (Go; [17]) and tactile/kinaesthetic factors (Gk) akin to Gv and Ga are potential candidates for inclusion in the CHC model of intelligence and there are others (e.g., gustatory). Some work in these areas has begun [2,3] but much more work with the tasks unique to each sensory modality is necessary. There is no doubt that recent developments in the experimental psychology of perception will need to be included in the study of individual differences.

It is important to understand that broad factors linked to sensory modalities include both elementary processes captured by the psychophysical tasks (i.e., assessment of threshold levels and sensory discrimination) and also more complex processes unique to the modality (e.g., tonal memory and tonal series completion tasks). These sensory modality processes tend to have lower correlations with processes from other sensory modalities and with the typical measures of “g”. Therefore, the amount of variance accounted for by the “g” factor is smaller than the amount of variance obtained from the analysis of HMP. For example, the average r = 0.29 correlation reported by Carroll [4] translates into about 35% of the total variance that can be attributed to the first principal component (i.e., “g”). This percentage of the total variance can be compared to the average of 23.8% obtained from five studies that included both HMP and sensory tasks listed in Table 2.1 in Stankov [10]. A lot more work needs to be done in relation to simple and complex cognitive processes tapped by different sensory modalities.

### 2.2. Biological Underpinnings: Not Much to Look Down to

While functional neuropsychological explanations of “g” in terms of mental efficiency [18], tuning-in of brain networks [19] and neural mechanisms postulated by the process overlap theory of Kovacs and Conway [20] or any other account mentioned by the commentators on their paper can still be used to explain such a small “g”, the role of neural networks that are involved in some of the broad factors of intelligence like Gf, Gc, Gv and Ga may turn out to be more interesting and important than “g” itself. In other words, brain processes associated with broad factors may be more tractable than those linked to “g” itself.

It should be noted that Kovacs and Conway [20] writings can be interpreted as an argument for adopting a “no-”g”” position. They claim that there is no such entity as a single “g” factor. Its appearance in statistical modeling is a reflection of it being an emergent, not a reflective, property. They hypothesize that statistical “g” is an emergent outcome of the sampled executive function abilities. A confirmatory modeling approach to the brain correlates of “g” by Kievit et al. [21] also concluded that “neuro-g” is a formative latent property determined by, rather than being the cause of or reflected by, neural measures. In agreement with this claim, Hampshire, Highfield, Parkin and Owen [22] reported that they found evidence to support the view that human intelligence is not unitary but, rather, is formed from multiple cognitive components and that “…there is little evidence for a higher-order intelligence factor” (p. 1223). Finally, despite considerable investment in research, it is worth noting that genetic studies of intelligence have failed to produce a breakthrough in the identification of genes responsible for the emergence of “g” (see [23]).

## 3. Emphasis on “g” is Restricting Research on Intelligence

Research on intelligence has been stifled because of the continuing emphasis on “g” factor and the use of a small number of measures, often only Raven’s Progressive Matrices (RPM) test, as its main assessment tools. In an effort to understand general intelligence factor in terms of cognitive psychology (in particular, working memory) and neuroscience, its broader psychometric aspects have been neglected.

For those working in the field of individual differences, the first exposure to the construct of working memory was through Hunt’s [24] paper. Some of us saw it as an example *par excellence* for the capacity interpretation of the cognitive basis of intelligence and being conceptually linked to the notion of attentional resources (see [25,26]). It has been studied extensively ever since and perhaps we now know as much as we need to know about working memory’s role in cognitive functioning and intelligence. Hunt’s [27] writings about the relationship between the attentional control-working memory complex and intelligence can be interpreted in a similar way. He points out that the research on that relationship falls into two categories. Many studies break down working memory into its components such as focusing attention, rehearsal, mental manipulations and the like. When correlated with IQ these component processes have frequently produced contradictory results and may now be seen as a waste of effort and time. A more useful approach in Hunt’s view is to treat information-processing components of working memory as a system and establish if the “…total system is functioning well enough, and quickly enough, to get the job done” (p. 170).

Psychometric work that has been restricted because of the emphasis on “g” refers both to psychological constructs and to the development of new measuring instruments. In the next sections some of the recent work that has a chance of being incorporated within the CHC framework will be outlined. The list of processes is conveniently grouped into those that have emerged either through the interaction with economics or from genuine psychological work. The list was compiled through my own interests in these areas. Undoubtedly, other researchers may be able to list many more equally important or even more neglected processes.

## 4. Economics and Decision-Making

Over the past three decades a rapprochement between economics and psychology has taken place and behavioral economics emerged as an academic discipline. It studies how cognitive, emotional and social processes affect economic decisions of individuals and institutions. This has replaced the idea of *homo economicus* who was conceived as a human that logically pursues self-interests (i.e., maximize his/her monetary benefits) and shows no regard for the welfare of others. Two areas—economic games and decision-making—have attracted the attention of researchers in individual differences.

### 4.1. Economic Games, Personality and Ability

There are a number of economic games available for research purposes today and most of them have several versions depending on the specific issues researchers are interested in. A couple of better known exemplars are prisoner’s dilemmas and dictator games. Most psychological research involving these games is related to personality characteristic. In the dictator game, for example, there are two players one of whom (i.e., the dictator) is given a certain amount of money and was told that (s)he can give some of that amount (it could be zero dollars) to the other player who, in turn, has to accept it. Under this condition, the economic theory assumes that all individuals act solely out of self-interest: the dictator should retain 100% of what (s)he has been given. However, in reality only 40% of people behave in the self-interested way while the remainder are generous and tend to share about 20% of the given amounts [28]. Researchers in individual differences are trying to link, for example, pro-social personality traits of agreeableness, honesty–humility and extraversion to generosity, egalitarianism and reciprocity behavior that can be assessed with economic games (see [29]). Overall, an evaluation of recent literature on the topic is likely to show that personality traits may be more relevant than ability in the prediction of behavior in two well-established major groups of economic games: social dilemmas and bargaining games.

Only occasionally have there been attempts to link cognitive ability to behavior displayed in economic games. For example, Yamagishi et al. [30] report that only 31 of 446 residents of a relatively wealthy Tokyo suburb met the behavioral definition of homo economicus. In the dictator and prisoner’s dilemma games these people apportioned the money endowed by the experimenter to themselves, leaving no share for their partners. These participants had high IQs. It would be probably worthwhile to link the other existing economic games to IQ as well. It would be even more interesting to design new economic games that may facilitate behavior along the lines of homo economicus and examine whether higher Gf and Gc participants will indeed become more “rich”. It is also apparent that high ability may play a role in some of the games in interaction with personality traits and outside of the homo economicus framework.

It appears that economic games can be used in three ways in studies of intelligence: (a) stripped of the aspects that are designed to assess behavior displayed in social interactions (e.g., benevolence) they can be used to develop items similar to those employed in typical IQ tests; (b) along the lines of the Yamagishi et al. [30] work, they can be used as predictors of real life achievements; and (c) economic games as such can be used as proxy criteria (i.e., “real life” approximations) for assessing predictive validity of the intelligence measures themselves.

### 4.2. Decisio- Making and Rationality

Research on individual differences has also benefitted from the study of decision-making. To date, the focus has been on cognitive biases and the argument has been that high IQ people are about equally likely to succumb to such biases as are the low IQ people. In other words, the correlation between intelligence and measures of cognitive biases is low (see [31]). Some of us are more rational and can avoid making wrong decisions while others may be more susceptible to errors irrespective of their IQ. People are described as being dysrational and one reason for it is the tendency to use heuristics rather than effortful processing in order to solve “tricky” problems—such people are cognitive misers. There is a long list of cognitive biases, some of which are based on empirical data and others derived from conceptual analyses and anecdotal evidence. The list includes anchoring bias, overconfidence bias, hindsight bias, base rate neglect, outcome bias and sunk cost effect among others.

The suggestion that rationality may be different from intelligence is appealing since, if proven, it can enrich our understanding of individual differences in cognitive processing. To deliver on this promise it will be necessary to employ the methodology and standards used in the typical studies of intelligence. This includes showing that measures of cognitive biases (a) have satisfactory reliability; (b) have correlations among themselves that are high enough to define a single (dys)rationality factor; and (c) have moderate correlation with measures of intelligence. Teovanović, Knežević and Stankov’s [32] results show that while requirement (a) is satisfied; (b) and (c) are not. In particular, correlations between the cognitive bias measures are too low (average r = 0.06) and the minimal number of factors that can be extracted from the correlational matrix of seven bias measures listed above is two, not one. In other words, the existence of a broad (dys)rationality factor is under question. Furthermore, in agreement with Stanovich’s [33] claims, the two cognitive bias factors have low correlations with Gf and Gc (average r = 0.17). Thus, it is doubtful whether they can be viewed as proper measures of cognitive functioning of the same ilk as those captured by tests of intelligence. The study of Aczel et al. [34] raises similar questions about the existence of a separate rationality factor.

There can be little doubt that research linking intelligence to the whole gamut of cognitive bias measures should continue since a different sampling of these measures may modify conclusions reached thus far. If future work confirms the findings to date, two interpretations of the cognitive bias measures may eventuate. One is in terms of the processes on the borderline between personality and abilities. According to this position, biases in decision-making reflect an amalgam of cognitive and non-cognitive processes. This is reminiscent of educational psychologists’ view of aptitude. For example, Snow [35] defines aptitudes broadly to accommodate cognitive, affective and conative characteristics. Similarly, Stanovich [33] suggests that cognitive biases may be linked to the dispositions toward actively open-minded thinking.

The other interpretation views cognitive biases as domain-specific activities perhaps of relevance to business and economic decision-making rather than general intelligence. Teovanović et al. [32] compare the status of the cognitive biases to the tests of neurological assessment that are not included in typical batteries for the measurement of intelligence in normal populations. They capture very specific processes and their predictive validity for real-life decision-making will yet need to be demonstrated.

## 5. Psychological Constructs

### 5.1. Emotional Intelligence: Almost There but More Work Needed

More than twenty years since the beginning of the work on emotional intelligence (EI) by Salovey and Mayer [36] there is evidence that processes captured by their MSCEIT battery of EI tests have become a candidate for the inclusion into the CHC framework. MacCann et al. [37] propose that Cattell-Horn-Carroll theory may be expanded to include EI as a second-stratum factor similar to fluid intelligence and visual processing. Legree et al. [38] used the same dataset but applied different scoring procedures and confirmed the existence of a broad EI factor. If replicated, perhaps with some additional ability EI tests, this would provide solid enough evidence for the acceptance of such a factor.

However, before incorporating EI into the CHC framework, there are further issues that will need to be addressed. A commonly applied procedure for the scoring of IE tests is consensus-based—i.e., for each item the score is calculated by comparing a participant’s answer to the key which is obtained from the answers provided by a sample of people who took the same test. For example, in the Faces test of EI, for each of four photographs of a face, participants rate (1 = *No emotion* to 5 = *Extreme emotion*) the extent to which the facial expression shows each of five different emotions. The score, referred to as the ‘proportion score’ or mean item endorsement ratio (ER), reflects the agreement between the participant’s ratings of the faces and the key contained within the scoring algorithm. A limitation of the consensual scoring procedure derives from the fact that background of the people who have provided the scoring key is unknown and comparisons (e.g., cross-cultural differences) in EI based on ER scores may be meaningless. But there is more.

Legree et al. ([38], Table 5) report correlations between the ER score and personality traits. It is well known that correlations between measures of cognitive abilities and personality are close to zero. In our own work, for example, the highest correlation of achievement and intelligence scores (r < 0.30) tends to be with the Openness to Experience measure which captures activities such as reading, going to the theatre and similar cultural events [9]. However, ER scores correlate unusually highly with the Big Five personality measure of Agreeableness (r = 0.42). This can be due to the consensual scoring procedure or, indeed, the likelihood that EI taps personality traits to an extent that its cognitive ability interpretation and therefore its place within the CHC may be open to question. Related claims have been made by Davies, Stankov and Roberts [39] and more recently by van der Linden et al. [40]. Addressing the status of EI within the CHC framework is arguably a more pressing task than examining the nature of “g” itself.

### 5.2. Complex Problem Solving Tasks

There are many opportunities for an expanded repertoire of cognitive assessment brought about through recent technological developments. Obviously, these include the use of virtual reality as a testing medium and computer games designed as cognitive training exercises, among others. Needless to say, with the advances in computer delivery, it became possible to employ measures of mental speed and to simultaneously deliver tests using a combination of sensory modalities (e.g., dual or competing tasks [41]). For those interested in personnel selection and assessment the relationship between computer literacy and cognitive abilities may also be an important object of study.

The first computer-based approaches that have been extensively correlated with measures of intelligence were introduced in the early 1980s. The attempts were made to develop computer simulations of the real life, usually managerial jobs (e.g., activities of the mayor of a small town) and the likes. German investigators carried much of the work in this area. Nowadays, these tasks are called complex problem solving tasks (CPS) or micro-worlds. Stadler et al. [42] credit Frensch and Funke [43] with the definition stating that these CPS tasks involve “(…) successful interaction with task environments that are dynamic (i.e., change as a function of the user’s interventions and/or as a function of time) and in which some, if not all, of the environment’s regularities can only be revealed by successful exploration and integration of the information gained in that process.” (p. 93). In these tasks the participant takes the role of managing a company—e.g., Furniture Factory—and is given a goal of, say, increasing the output of some kind. Following a particular decision, a computer algorithm provides feedback and the participant has to figure out how different elements of the task interact and lead to an improvement in performance. Clearly, CPS tasks have better face validity for predicting performance in managerial jobs than typical tests of intelligence. Stadler et al. [42] report the outcomes of a meta-analysis of 47 studies and a total sample size of 13,740 participants that revealed a reasonable correlation (r = 0.43) between CPS and intelligence. Obviously, there is a sufficient amount of variance in common and complex problem solving tasks can be seen as plausible replacements for the typical intelligence tests in some situations. Their predictive validity is likely to be at least similar to that of typical reasoning and intelligence tests.

The work on complex problem solving does not seem to have been affected much by the emphasis on “g” in intelligence research. However, it would be useful to see the integration of this work into the hierarchical models of intelligence. Two issues are of particular interest and, although both have been addressed in a hitherto unpublished PhD work of Ryan [44], they do not seem to have been sufficiently explored in the extant literature on complex problem solving. First, it is not known whether the correlations between different CPS tasks define a separate, perhaps akin to the EI, broad factor. Ryan’s work was based on N = 296 participants who were given three tasks: Furniture Factory (FF), Taylorshop (T) and Forestry System (FS) along with a battery of 16 measures of Gf, Gc and broad visualization, Gv. The average correlation between the three CPS tasks was 0.22 (r_FF,T_ = 0.30, r_FF,FS_ = 0.27, r_T,FS_ = 0.10), suggesting that there may not exist a strong common process underlying complex problem solving. Second, even though different measures of intelligence have been correlated with the CPS tasks, it is not known whether these tasks are better measures of Gf or Gc and whether they may capture some other broad factors from the CHC theory. Ryan’s [44] findings indicate that while both Gf (average r = 0.26) and Gc (average r = 0.34) are implicated in CPS, Gc appears to be a somewhat stronger predictor of CPS performance. Clearly, more work is needed both in order to find out if there are common processes underlying CPS tasks and about their place within the hierarchical models of intelligence.

## 6. Summary and Discussion

All four topics listed above will need further work before they can be included within the CHC theory or, as it may happen, placed elsewhere within the discipline of psychology. The findings with *emotional intelligence* are the closest to being acceptable. The hierarchical structure of EI needs to be replicated and the scoring needs to be examined to reduce unusual correlation with personality traits. More work will need to be done with the *complex problem solving* tasks. Commonalities between the different versions of these tasks will have to be established and it will be necessary to find out if they define a broad CPS factor or are simply to be seen as additional markers for established factors like Gf and Gc.

Interaction between economics and psychology has been interesting and fruitful but it is still unclear whether it will lead to major additions or modification of the CHC framework. The work on *decision-making* and *rationality* has produced new measures of cognitive biases. These reflect errors in reasoning. It is possible that some of these bias measures will be related to measures of intelligence and, in particular, to the Gc processes but the evidence available now indicates low correlations. Consequently, rationality may indeed be different from intelligence but in order to make definite claims to this effect it will be necessary to spell out the nature of the evidence in support of such a conclusion. How can rationality stand on its own when its indicants measure specific processes that share little among themselves? Finally, some *economic games* may turn out to be linked to cognitive processing but for the time being they seem to be best described as outcome/criterion measures that are related to individual differences in personality and much less to cognitive abilities.

Even though I have treated the developments within the four domains as independent, there is a good reason to expect that some cross-fertilization between researchers working in these domains will take place in future. The first signs of this have emerged since Rudoph et al. [45] have employed confidence ratings—i.e., typical measures used for assessing overconfidence bias and metacognitive monitoring—in a new study of CPS. Their finding was that confidence in CPS explained more than 50% of variance in performance. These effects were reduced but remained substantial when they controlled for reasoning. The conclusion is that their results indicate that confidence judgments as indicators of metacognitive monitoring in CPS are substantially linked to successful CPS performance.

Trying to place new measures and constructs within the CHC framework is important particularly because they may be closely aligned with broad CHC factors. For example, from among the four areas considered in this paper emotional intelligence, decision-making and economic games may be related to Gc whereas Stadler et al. [42] show that complex decision-making is also related to reasoning and Gf. In other words, some of these new areas may be more dependent on participants’ knowledge and education than other skills and abilities.

Apart from the need to also explore further the role of tasks specific to sensory modalities there are at least two other areas of relevance to the study of intelligence our team has been involved with that have been neglected because of the emphasis on “g”. Both areas are related to educational interventions. One is the attempt to use training of cognitive performance in an effort to increase intelligence. The second is related to the elimination of impediments (e.g., test anxiety) and the increased use of non-cognitive facilitators (e.g., calibration of self-beliefs) of cognitive performance (see [9]).

It is important to keep in mind that the existence of “g” is not being questioned. Inclusion of the specific sensory modality tests into the analyses tends to reduce the strength of “g”. Cognitive bias measures are likely to have the same impact. However, the other three constructs listed above will tend to leave the percentage of variance attributable to “g” unchanged. Overall, the role of “g” is certainly lower than what it is commonly believed to be.

I am also of the opinion that the focus on “g” has affected the work on other interesting and relevant cognitive constructs. How else can we explain the fact that initial work on all four domains mentioned above was started more than ten years ago and no firm link to CHC theory has been established? In our studies of intelligence we need to be more open-minded and explore the cognitive domain in its entirety and be ready to accept suggestions from the disciplines other than our own. For example, people working in the areas of business and management as well as the military have become interested in collaborative group work and there have been papers reporting on the collective intelligence factor in group performance (see [46]; but see also [47]). We also need to be prepared to take full advantage of the technical facilities at our disposal today. Using paper-and-pencil versions of our tests appears to be working but there are so many other options open to us that should be more actively explored. Innovations in assessment that go beyond simple translation of these tests into their digitized equivalents hold promise of considerably enhancing predictive validity of our measures and discovering new psychological processes that play a role in our lives. Finally, neuropsychological bases of cognitive processing need to be understood but the emphasis on “g” can take us away from the study of neural networks corresponding to the important lower-stratum processes within the hierarchy. It seems reasonable to assume that identifying neural networks linked to, say, Gc will be easier than finding networks linked to both Gf and Gc (i.e., “g”) as a single entity.

To move forward we need to accept that intelligence refers to the study of individual differences in all cognitive abilities. We should be open to considering every task and process that can be classified as such. We also need to accept that tasks on the borderline between cognitive and non-cognitive domains may be relevant as well. Our goal should be to build a taxonomy of these tasks using multivariate statistical procedures on our disposal.
